# Assessment of Spoilage Bacterial Communities in Food Wrap and Modified Atmospheres-Packed Minced Pork Meat Samples by 16S rDNA Metagenetic Analysis

**DOI:** 10.3389/fmicb.2019.03074

**Published:** 2020-01-21

**Authors:** Emilie Cauchie, Laurent Delhalle, Bernard Taminiau, Assia Tahiri, Nicolas Korsak, Sophie Burteau, Papa Abdoulaye Fall, Frédéric Farnir, Ghislain Baré, Georges Daube

**Affiliations:** ^1^Department of Food Sciences, Fundamental and Applied Research for Animals & Health (FARAH), Faculty of Veterinary Medicine, University of Liège, Liège, Belgium; ^2^Quality Partner sa, Liège, Belgium

**Keywords:** minced meat, metagenetics, spoilage bacteria, modified atmosphere packaging, food wrap packaging

## Abstract

Although several studies have focused on the dynamics of bacterial food community, little is known about the variability of batch production and microbial changes that occur during storage. The aim of the study was to characterize the microbial spoilage community of minced pork meat samples, among different food production and storage, using both 16S rRNA gene sequencing and classical microbiology. Three batches of samples were obtained from four local Belgian facilities (A–D) and stored until shelf life under food wrap (FW) and modified atmosphere packaging (MAP, CO_2_ 30%/O_2_ 70%), at constant and dynamic temperature. Analysis of 288 samples were performed by 16S rRNA gene sequencing in combination with counts of psychrotrophic and lactic acid bacteria at 22°C. At the first day of storage, different psychrotrophic counts were observed between the four food companies (Kruskal-Wallist test, *p*-value < 0.05). Results shown that lowest microbial counts were observed at the first day for industries D and A (4.2 ± 0.4 and 5.6 ± 0.1 log CFU/g, respectively), whereas industries B and C showed the highest results (7.5 ± 0.4 and 7.2 ± 0.4 log CFU/g). At the end of the shelf life, psychrotrophic counts for all food companies was over 7.0 log CFU/g. With metagenetics, 48 OTUs were assigned. At the first day, the genus *Photobacterium* (86.7 and 19.9% for food industries A and C, respectively) and *Pseudomonas* (38.7 and 25.7% for food companies B and D, respectively) were dominant. During the storage, a total of 12 dominant genera (>5% in relative abundance) were identified in MAP and 7 in FW. *Pseudomonas* was more present in FW and this genus was potentially replaced by *Brochothrix* in MAP (two-sided Welch’s *t*-test, *p*-value < 0.05). Also, a high Bray-Curtis dissimilarity in genus relative abundance was observed between food companies and batches. Although the bacteria consistently dominated the microbiota in our samples are known, results indicated that bacterial diversity needs to be addressed on the level of food companies, batches variation and food storage conditions. Present data illustrate that the combined approach provides complementary results on microbial dynamics in minced pork meat samples, considering batches and packaging variations.

## Introduction

Meat and meat products are highly perishable, with colonization and development of a variety of microorganisms, especially bacteria. This is due to complex nutrient-rich environment with chemical and physical conditions favorable to bacterial development (Nychas et al., 2008; Pennacchia et al., 2009; Chaillou et al., 2015; Garnier et al., 2017). Moreover, minced meat can be contaminated by different types of microorganisms from several sources, such as raw materials, equipment, environment and handling involved in the production process. Abiotic factors (temperature, gaseous atmosphere, pH, NaCl levels, etc.) can also select certain bacteria (Mann et al., 2016; Stellato et al., 2016; Rouger et al., 2018). However, it is well known that richness and abundance of microbiota present in food products, and especially meats, play an important role in the microbial safety and the shelf life of the products (Zhao et al., 2015; Pinu, 2016). Microbial growth on meat to unacceptable levels and the various metabolic activities contribute to its deterioration by altering the structure, color and flavor of the meat (Mann et al., 2016). This leading to a reduction in food quality to the point of not being edible for human consumption (Holm et al., 2013; Silbande et al., 2016; Stellato et al., 2016), with alterations in the sensorial qualities of the product, particularly the aspect, with discoloration and gas production, and the presence of an off-odors and off-flavors (Stoops et al., 2015). Thus, food spoilage is problematic for two main reasons: first, it renders food unfit for human consumption and, secondly, it results in significant economic losses (Dalcanton et al., 2013; Pinter et al., 2014; Den Besten et al., 2017).

As mentioned by Benson et al. (2014), the microbial population that colonizes and ultimately spoils minced pork meat is highly variable, depending on which groups of microbial taxa the product has been exposed to and perhaps even the order in which they are encountered. Using traditional cultivation methods, the microbial composition and diversity in fresh meat have been widely investigated (Zhao et al., 2015), but it is well known that traditional identification and culture-based methods for pathogens or food spoilage microbes are time-consuming (Pinu, 2016). Moreover, ecological studies at the genus-species level are required because the same storage conditions may affect differently the species in the same groups of bacteria (Pennacchia et al., 2011; Stoops et al., 2015), and because not all the members of this microbiota contribute to food spoilage. Several studies in meat microbiology have established that spoilage is caused only by a dominated fraction of the initial microbial association (Nychas et al., 2008). These spoilage microorganisms have been designated as Ephemeral/Specific Spoilage Organisms (E(S)SOs) (Benson et al., 2014; Zotta et al., 2019). Therefore, as discussed by De Filippis et al. (2013), the concept of succession of spoilage-related microbial groups is very important, and many studies have been performed to investigate the dynamics and changes of the meat microbiota during storage.

Developed during the last decades, the next generation sequencing methodologies provide a powerful tool to study microbial community structure and composition shifts at different stages of ripening, allowing the detection of minor bacterial populations (Riquelme et al., 2015), at variable taxonomic depth (Pothakos et al., 2014; Chaillou et al., 2015; Parente et al., 2016). The introduction of molecular methods, especially culture-independent approaches, have contributed to the exploration of various food microbiota (Galimberti et al., 2015; Pinu, 2016; Garofalo et al., 2017; Parlapani et al., 2018), as for beverages (Elizaquivel et al., 2015), vegetables (Lee et al., 2017; Gu et al., 2018; Liu et al., 2019), and for dairy (Nalbantoglu et al., 2014; Riquelme et al., 2015; Ceugniez et al., 2017; Porcellato et al., 2018), seafood (Li et al., 2018; Parlapani et al., 2018; Silbande et al., 2018), and meat products (Cocolin et al., 2004; Pennacchia et al., 2011; Nieminen et al., 2012; Benson et al., 2014; Greppi et al., 2015; Polka et al., 2015; Stoops et al., 2015; Zhao et al., 2015; Delhalle et al., 2016; Mann et al., 2016; Carrizosa et al., 2017; Cauchie et al., 2017; Kaur et al., 2017; Korsak et al., 2017; Peruzy et al., 2019; Vester Lauritsen et al., 2019), in order to assess the microbial levels and diversity of food and food products (Nieminen et al., 2012; Pothakos et al., 2014; Lee et al., 2017; Rouger et al., 2018). The interest of this method to characterize the dominant spoilage bacteria in pork meat and meat products was also described (Andritsos et al., 2012; Mann et al., 2016; Raimondi et al., 2018; Li et al., 2019; Peruzy et al., 2019).

In this context, the aim of the present study was to assess the microbial spoilage community and dynamics of minced pork meat samples, among different conditions of production and food storage, using both 16S rRNA gene sequencing and classical microbiology.

## Materials and Methods

### Sampling

Fresh minced pork meat (MPM) samples packed with a food wrap film were obtained from four local small and medium-sized Belgian manufacturers (food companies A, B, C, and D) at the day of the production, corresponding to the day of slaughtering. Three batches for each manufacturer were used, with a 1-week interval between sampling (Supplementary Figure S1).

According to the recipe MPM is composed of 100% minced pork meat (70% lean, 30% fat), no salt, no spices, no additives, no eggs and no sugar are added. At the day of the production, the water activity of this product was 0.98 ± 0.02 and the pH value was 5.80 ± 0.05 (*n* = 12). pH of the homogenized samples (5 g in 45 ml of KCl) was measured with a pH meter (Knick 765 Calimatic, Allemagne). The water activity was measured for homogenized samples on the basis of the relative humidity measurement of the air balance in the micro enclosure at 25 ± 0.4°C (Thermoconstanter TH200, Novasina, Switzerland).

Minced pork meat samples were packed (100 g), in triplicate, in two different types of non-sterile packaging.

The first packaging concerns a tray (187 × 137 × 36, polyester 10 μm, homo-polymer polypropylene 50 μm, NutriPack, France) under modified atmosphere (MAP, CO_2_ 30%/O_2_ 70% ± 0.1%) (Olympia V/G, Technovac, Italy) using packaging wrap (PP/EVOH/PP) with random gas measurements (CheckMate 3, Dansensor, France).

The second packaging concerns a tray (175 × 135 × 22, polystyrene) under food wrap packing (FW) using cling film (Clinofilm).

### Food Storage

According to the requirements for implementing microbiological tests of chilled perishable and highly perishable foodstuffs (AFNOR, 2010, NF V01-003), MPM samples were stored during 3 days of shelf life under FW, and during 6 days under MAP packaging, at constant and dynamic temperature: at (i) 2°C (± 1°C), (ii) 8°C (± 1°C), (iii) 12°C (±1°C), and (iv) for a third of the shelf life at 2°C and for the rest of the shelf life at 8°C (2/8°C ± 1°C), in climatic chambers (Sanyo MIR 254).

Samples were analyzed at the first day of inoculation (day 0) and at the last day of storage (day 3 in FW and day 6 in MAP, n = 288) (Supplementary Figure S1).

### Plate Count Enumeration

Twenty-five grams of product were randomly collected from the trays at the surface and at depth, without homogenization, and put into a Stomacher bag with a mesh screen liner (80 μm pore size) (Biomérieux, Basingstoke, England, ref 80015) under aseptic conditions. Buffered peptone water (BPW, 10 g/L peptone, 5 g/L sodium chloride, #3564684, Bio-Rad, Marnes-la-Coquette, France) (225 mL) was automatically added to each bag (Dilumat, Biomérieux, Belgium) and the samples were homogenized for 2 min in a Stomacher (Bagmixer, Interscience, France). From this primary suspension, decimal dilutions in maximum recovery diluent (10 g/L peptone, 8.5 g/L sodium chloride, #CM0733, Oxoid, Hampshire, England) were prepared for microbiological analysis, and 0.1 mL aliquots of the appropriate dilutions were plated onto media for each analysis (Spiral plater, DW Scientific, England). Total viable counts (TVC) for the aerobic psychrotrophic flora were performed on plate count agar (PCA agar, #3544475, Bio-Rad, Marnes-la-Coquette, France), and for the lactic acid bacteria (LAB) on de Man, Rogosa and Sharpe (MRS agar, #CM0361, Oxoid, Hampshire, England), after incubation at 22°C (Pothakos et al., 2014) for 72 h (model 1535 incubator, Shel Lab, Sheldon Manufacturing Inc., United States).

### DNA Extraction and 16S rDNA Amplicon Sequencing

Bacterial DNA was extracted from each primary suspension, previously stored at −80°C, using the DNEasy Blood and Tissue kit (QIAGEN Benelux BV, Antwerp, Belgium) following the manufacturer’s recommendations. The resulting DNA extracts were eluted in DNAse/RNAse free water and their concentration and purity were evaluated by means of optical density using the NanoDrop ND-1000 spectrophotometer (Isogen, Sint-Pieters-Leeuw, Belgium). DNA samples were stored at −20°C until used for 16S rDNA amplicon sequencing.

PCR-amplification of the V1-V3 region of the 16S rDNA library preparation were performed with the following primers (with Illumina overhand adapters), forward (5′-GAGAGTTTGATYMTGGCTCAG-3′) and reverse (5′-ACCGCGGCTGCTGGCAC-3′). Each PCR product was purified with the Agencourt AMPure XP beads kit (Beckman Coulter; Pasadena, CA, United States) and submitted to a second PCR round for indexing, using the Nextera XT index primers 1 and 2. Thermocycling conditions consisted of a denaturation step of 4 min at 94°C, followed by 25 cycles of denaturation (15 s at 94°C), annealing (45 s at 56°C) and extension (60 s at 72°C), with a final elongation step (8 min at 72°C). These amplifications were performed on an EP Mastercycler Gradient System device (Eppendorf, Hamburg, Germany). The PCR products of approximately 650 nucleotides were run on 1% agarose gel electrophoresis and the DNA fragments were plugged out and purified using a Wizard SV PCR purification kit (Promega Benelux, Leiden, Netherlands). After purification, PCR products were quantified using the Quanti-IT PicoGreen (ThermoFisher Scientific, Waltham, MA, United States) and diluted to 10 ng/μL. A final quantification, by quantitative (q)PCR, of each sample in the library was performed using the KAPA SYBR^®^ FAST qPCR Kit (KapaBiosystems, Wilmington, MA, United States) before normalization, pooling and sequencing on a MiSeq sequencer using V3 reagents (Illumina, San Diego, CA, United States).

### Bioinformatics Analysis

The 16S rRNA gene sequence reads were processed with MOTHUR (Schloss et al., 2009). The quality of all sequence reads was denoised using the Pyronoise algorithm implemented in MOTHUR. The sequences were checked for the presence of chimeric amplification using ChimeraSlayer (developed by the Broad Institute)^1^. The obtained read sets were compared to a reference data-set of aligned sequences of the corresponding region derived from the SILVA database of full-length rRNA gene sequences (version v1.2.11)^2^ implemented in MOTHUR (Pruesse et al., 2012; Pothakos et al., 2014; Cauchie et al., 2017). The final reads were clustered into operational taxonomic units (OTUs), using the nearest neighbor algorithm using MOTHUR with a 0.03 distance unit cut off. A taxonomic identity was attributed to each OTU by comparison to the SILVA database, using an 80% homogeneity cut off. As MOTHUR is not dedicated to the taxonomic assignment beyond the genus level, all unique sequences for each OTU were compared to the SILVA data-set 111, using a BLASTN algorithm. For each OTU, a consensus detailed taxonomic identification was given based upon the identity (<1% mismatch with the aligned sequence) and the metadata associated with the best hit (validated bacterial species or not) (Delcenserie et al., 2014; Cauchie et al., 2017).

### 16S rDNA Data Analysis

A correcting factor for 16S rDNA gene copy numbers was applied for any taxon *i* (Eq. 1) (Kembel et al., 2012; Louca et al., 2018).

(Eq. 1) Ai = NkCi

Where *A*_*i*_ is the real abundance of 16S genes from the taxon in the sample, *N*_*k*_ is the number of reads for the taxon in the sample *k*, and *C*_*i*_ is determined by the genomic 16S copy number of that taxon. To obtain each gene copy number, Ribosomal RNA Database (rrnDB) (Stoddard et al., 2015) and EzBioCloud database (Yoon et al., 2017) were used.

Then, to compare the relative abundance of OTUs, the number of reads of each taxon were normalized (*Nr*_*i*_) as described by Chaillou et al. (2015). Reads counts of each taxon *i* in the sample *k* were divided by a sample-specific scaling factor *(Si)* (Eq. 2) (Fougy et al., 2016; Rouger et al., 2018):

(Eq. 2) Nri = AiSk

Where *A*_*i*_ is the real abundance of 16S genes from that taxon obtained with a correcting factor for 16S rDNA gene copy numbers, *S*_*k*_ is the normalization factor associated with sample *k*.

The sample-specific scaling factor was calculated by (Eq. 3):

(Eq. 3) Sk = Tkme

Where *S*_*k*_ is the sample-specific scaling factor associated with sample *k*, *T*_*k*_ is the number of total reads in the sample *k*, *m*_*e*_ is the median value of total reads for all the samples of the dataset. Reads counts of all samples were then transformed into a percentage of each OTUs.

All biosample raw reads were deposited at the National Center for Biotechnology Information (NCBI) and are available under de BioProject ID PRJNA551357. The raw data supporting the conclusions of this manuscript will be made available by EC to any qualified researcher.

### Statistical Analysis

#### Statistical Analysis on Microbiological Results

Non-parametric statistical tests were used to compare the classical microbiology result between samples taken on the day of production and at the end of shelf life for a same temperature. With the help of R software (R Core Team, 2016), Kruskal-Wallis test was performed to make a comparison between the food industries on a certain day (i.e., day 0 or day 3) (stats package, kruskal.test function). An Analysis of Covariance (ANCOVA) was also performed to evaluate the interactions between the storage conditions and the food origin on psychrotrophic counts (FactoMineR package, AovSum function). All tests were considered as significant for a *p*-value of < 0.05.

#### Statistical Analysis on 16S rDNA Results

Alpha diversity for each sample was evaluated by richness estimation (Chao1 estimator), microbial biodiversity (inverse of the Simpson index, coverage), and the population evenness (Simpson evenness) using MOTHUR (version 1.40.5)^3^ (Riquelme et al., 2015; Zhao et al., 2015). Rarefaction curves were calculated for all samples to ensure that sequencing depth was sufficient: OTUs identified were plotted as a function of sequences obtained per sample. High diversity coverage was achieved with all curves reaching asymptotes from 3000 reads (Supplementary Figure S2). Using Explicet, alpha and beta diversity indices were also calculated with bootstrapped sequencing data^4^ (Robertson et al., 2013; Mann et al., 2016).

Beta-diversity was assessed with Explicet using the Bray-Curtis index on a 0-1 scale.

Using STAMP (v2+) software^5^, a 2-sided Welch’s *t*-test was performed on metagenetic results and confidence intervals were calculated according to the Newcombe-Wilson method. A Principal Component Analysis (PCoA) was also applied to classify and cluster samples according to the identified OTUs for the two packaging (Tukey-Kramer test in conjunction with an ANOVA) (Parlapani et al., 2018). The differences were considered significant for a corrected *p*-value of less than 0.05 (Parks et al., 2014).

## Results

### Microbiological Analysis

As expected, psychrotrophic and lactic aerobic counts increased during the shelf life with increasing the temperature (Tables 1, 2).

**TABLE 1:** Results of psychrotrophic aerobic counts in minced pork meat samples according to the origin, the food packaging and the temperature of storage.

Industries/packaging	Day 0	End of the shelf life (day 3)			
		
		2°C	8°C	12°C	2/8°C
**FW**					
A	5.6 ± 0.1	6.5 ± 0.6	8.3 ± 0.4^∗^	8.3 ± 0.5^∗^	8.3 ± 0.3^∗^
B	7.5 ± 0.4	7.5 ± 0.4	8.3 ± 0.0^∗^	8.3 ± 0.2^∗^	8.3 ± 0.9^∗^
C	7.2 ± 0.4	7.3 ± 0.5	7.8 ± 0.0	7.8 ± 0.2	7.6 ± 1.3
D	4.2 ± 0.4	4.6 ± 0.2	7.2 ± 0.2^∗^	8.3 ± 0.0^∗^	6.6 ± 0.2^∗^
Kruskal-Wallis test	9.43 (0.02)°	8.74 (0.03)°	9.02 (0.03)°	5.71 (0.13)	9.68 (0.02)°
**MAP**					
A	5.6 ± 0.1	6.5 ± 0.1^∗^	7.9 ± 0.1^∗^	8.3 ± 0.3^∗^	7.9 ± 0.2^∗^
B	7.5 ± 0.4	7.9 ± 0.1	8.3 ± 0.0^∗^	8.3 ± 0.0^∗^	8.3 ± 0.0^∗^
C	7.2 ± 0.4	7.5 ± 0.2	7.6 ± 0.1	8.3 ± 0.1^∗^	7.8 ± 0.6
D	4.2 ± 0.4	5.2 ± 0.3^∗^	7.9 ± 0.1^∗^	8.1 ± 0.1^∗^	7.2 ± 0.1^∗^
Kruskal-Wallis test	9.43 (0.02)°	10.39 (0.02)°	9.68 (0.02)°	3.45 (0.33)	8.94 (0.03)°

*Values given as log CFU/g (mean ± SD, *n* = 3) at 2, 8, 12, and 2/8°C. FW (food wrap packaging), MAP (modified atmosphere packaging),° significant Kruskal-Wallis value (*p* < 0.05) with *p*-value between bracket, ^∗^significant Wilcoxon value (*p* < 0.05).*

**TABLE 2:** Results of lactic aerobic counts in minced pork meat samples according to the origin, the food packaging and the temperature of storage.

Industries/packaging	Day 0	End of the shelf life (day 6)			
		
		2°C	8°C	12°C	2/8°C
**FW**					
A	5.2 ± 0.2	6.4 ± 0.4	7.8 ± 0.1^∗^	7.8 ± 0.2^∗^	7.4 ± 0.2^∗^
B	5.5 ± 0.6	5.5 ± 0.5	7.1 ± 0.3^∗^	7.9 ± 0.2^∗^	6.8 ± 0.4^∗^
C	5.2 ± 0.7	6.7 ± 0.2^∗^	7.4 ± 0.1^∗^	7.6 ± 0.1^∗^	7.0 ± 0.2^∗^
D	3.5 ± 0.2	4.4 ± 0.3^∗^	5.9 ± 0.4^∗^	7.5 ± 0.1^∗^	5.1 ± 0.3^∗^
Kruskal-Wallis test	8.90 (0.04)°	9.15 (0.03)°	9.67 (0.02)°	7.62 (0.05)	8.44 (0.04)°
**MAP**					
A	5.2 ± 0.2	7.1 ± 0.2^∗^	8.0 ± 0.18^∗^	8.2 ± 0.09^∗^	8.2 ± 0.09^∗^
B	5.5 ± 0.6	6.6 ± 0.6^∗^	7.8 ± 0.21^∗^	7.7 ± 0.16^∗^	7.8 ± 0.15^∗^
C	5.2 ± 0.7	7.3 ± 0.2^∗^	7.6 ± 0.06^∗^	7.9 ± 0.09^∗^	7.5 ± 0.07^∗^
D	3.5 ± 0.2	5.2 ± 0.4^∗^	7.5 ± 0.07^∗^	7.8 ± 0.03^∗^	6.8 ± 0.24^∗^
Kruskal-Wallis test	8.90 (0.04)°	8.44 (0.04)°	9.05 (0.03)°	8.27 (0.04)°	9.45 (0.02)°

*Values given as log CFU/g (mean ± SD, *n* = 3) at 2, 8, 12, and 2/8°C. FW (food wrap packaging), MAP (modified atmosphere packaging),° Significant Kruskal-Wallis value (*p* < 0.05) with *p*-value between bracket, ^∗^significant *t*-student value (*p* < 0.05).*

Compared to the TVC values, LAB counts showed highest results for food industries A and D.

At day 0, different microbiological counts were observed between food companies for TVC (Kruskal-Wallis test, *H* = 9.43, *p*-value = 0.02) and for LAB (Kruskal-Wallis tests, *H* = 8.90, *p*-value = 0.04). The lowest psychrotrophic populations were observed for food industries D (4.2 ± 0.4 log CFU/g) and A (5.6 ± 0.1 log CFU/g), whereas minced pork meat samples from B to C showed the highest results (7.5 ± 0.4 and 7.2 ± 0.4 log CFU/g, respectively).

At the end of the shelf life, the natural logarithm of the TVC for all food companies was over 7.0 log CFU/g. At this time, the Analysis of Covariance revealed also a significant effect of the food companies (*p*-value = 0.00000998) and the temperature of storage (*p*-value = 0.00000095) on microbial total counts. Psychrotrophic counts seems also to be influenced by the interaction of the food industry and the temperature (*p*-value = 000442), but not by others interactions terms (*p*-value > 0.05).

### Carbon Dioxygen Production

**FIGURE 1:**
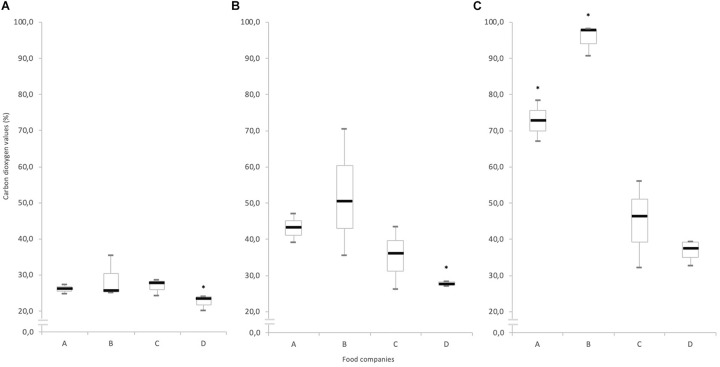
Box plots show the carbon dioxide measurements at the end of the shelf life, for the four food companies (A–D) at **(A)** 2°C, **(B)** 8°C, and **(C)** 12°C. The boxes represent the interquartile range between the first (Q1) and the third (Q3) quartiles; the vertical black line insides the box is the median obtained from the three batches analyzed by food industries; the two dotted line is the difference of 25% below the Q1 or above the Q3. The presence of stars indicated that samples deviated significantly from the carbon dioxide value at day 0 (30.0 ± 0.1%).

As shown in Figure 1, carbon dioxygen values increased with highest temperatures, except for the food companies C and D which shown relatively stable measurements. Results at 2/8°C are not shown in this paper.

### Alpha Diversity of Bacteria With 16S rDNA Amplicon Sequencing

Over 4,200 reads per sample were generated with pyrosequencing. In total, 48 mains OTUs were assigned. The number of OTUs, the bacterial diversity, richness estimators and coverage are presented in Supplementary Tables S1–S3). The highest number of identified species was encountered for the food industries C and D.

### Bacterial Communities at the Family and Genus Levels

**FIGURE 2:**
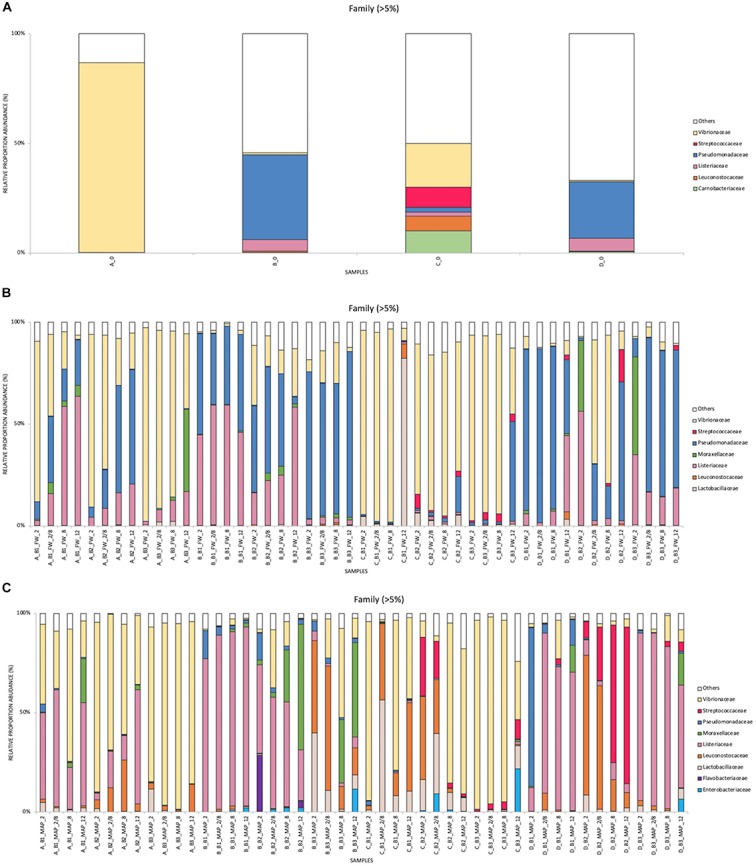
Cumulated histograms of the relative abundance (%) of taxa and the dynamics of the bacterial community identified by metagenetics at Family levels, during cold storage of minced pork meat in relation to the food packaging and the origin of samples (food companies and batches). **(A)** food samples analyzed at day 0 for the four companies (A–D), **(B)** storage in FW (food wrap) packaging, **(C)** storage in MAP (modified atmosphere) packaging. At Family levels, the taxa representing <5% in relative abundance were merged in the category of “Others”. Legend: batch 1 (B1), batch 2 (B2), batch 3 (B3), at 2°C (2), at 8°C (8), at 12°C (12), and for a third of the shelf life at 2°C and for the rest of the shelf life at 8°C (2/8).

**FIGURE 3:**
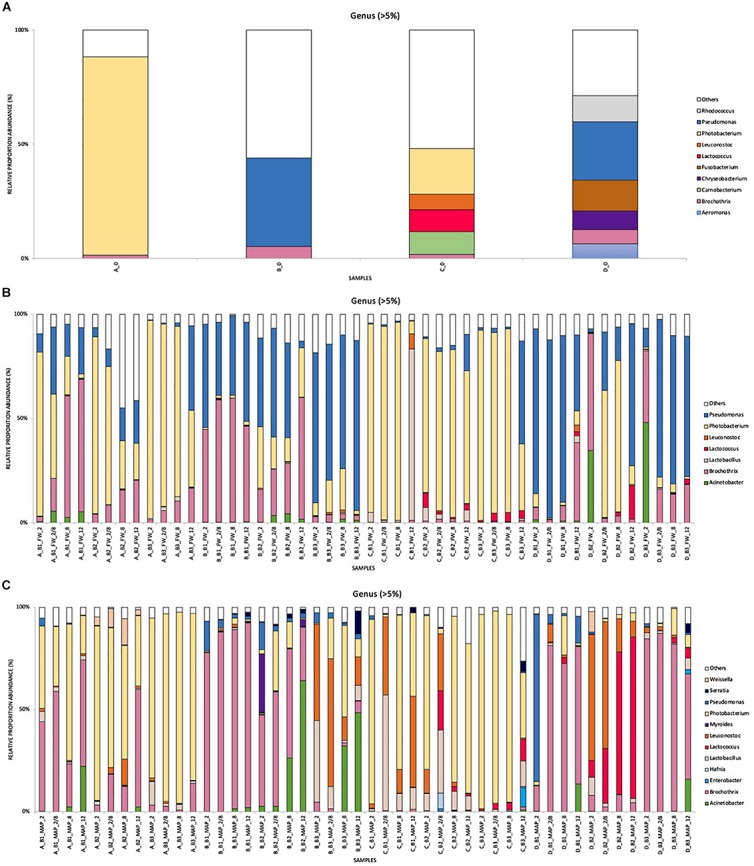
Cumulated histograms of the relative abundance (%) of taxa and the dynamics of the bacterial community identified by metagenetics at Genus levels, during cold storage of minced pork meat in relation to the food packaging and the origin of samples (food companies and batches). **(A)** food samples analyzed at day 0 for the four companies (A–D), **(B)** storage in FW (food wrap) packaging, **(C)** storage in MAP (modified atmosphere) packaging. At Genus levels, the taxa representing <5% in relative abundance were merged in the category of “Others”. These data including the relative abundance of sequences are also summarized in Supplementary Tables S4–S6. Legend: batch 1 (B1), batch 2 (B2), batch 3 (B3), at 2°C (2), at 8°C (8), at 12°C (12), and for a third of the shelf life at 2°C and for the rest of the shelf life at 8°C (2/8).

The relative abundance results obtained by metagenetics analysis (expressed in%) in FW and MAP packaging at Family (Figure 2) and Genus (Figure 3) levels (>5%) are represented in cumulated histograms for all samples. These data including the relative abundance of sequences are also summarized in Supplementary Tables S4–S6). The taxa representing <5% in relative abundance were merged in the category of “Others.” “Others” in FW are mainly composed by the genera *Bacillus*, *Carnobacterium*, *Enterococcus*, *Hafnia*, *Myroides*, *Rahnella*, *Staphylococcus*, *Serratia*, *Streptococcus*, *Weissella* and *Xanthomonas* in FW. While it concerns *Bacillus*, *Carnobacterium*, *Enterococcus*, *Hafnia*, *Rahnella*, *Staphylococcus*, *Streptococcus* and *Xanthomonas* in MAP. Full data on taxa found in high (>5%) and low (<5%) frequencies will be made available by EC to any qualified researcher.

According to Figures 2, 3, the food companies show a high variability in the distribution of read percentages at day 0. At this time, the genus *Photobacterium* is the most represented for A and C (86.7 and 19.9%, respectively), while it concerns the genus *Pseudomonas* for the industries B and D (38.7 and 25.7%, respectively).

At the end of the shelf life, a total of 12 genera were identified as dominant (taxa representing more than 5% in relative abundance) in MAP and only seven genera in FW. These seven genera are all identical to those found in MAP.

For all samples, the percentage of “unassigned” reads was relatively low (7.1 ± 3.7).

### Effect of the Food Packaging on the Bacterial Communities

**FIGURE 4:**
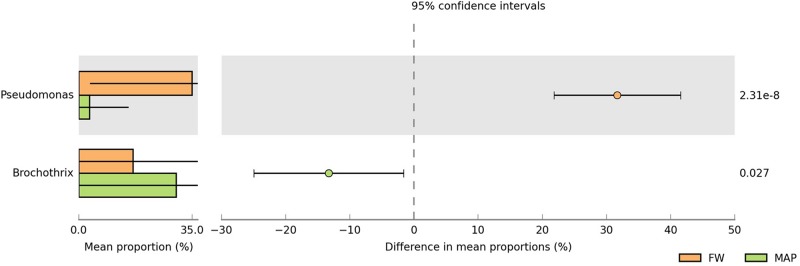
Extended bar plot showing the bacterial populations whose mean relative abundance differed between food wrap (FW) and modified atmosphere (MAP) packaging at genus scale. The relative abundance and the difference in mean proportions are illustrated for the statistically different taxa (*p* < 0.05).

However, although dominant genera were identified across all samples, the two different types of packaging were characterized by different microbiota, with only some genera in common (Supplementary Figure S3). At the end of the shelf life, *Pseudomonas* was more present in FW and this genus was potentially replaced by *Brochothrix* in the MAP packaging (Welch’s *t*-test, *p*-value < 0.05) (Figure 4).

**FIGURE 5:**
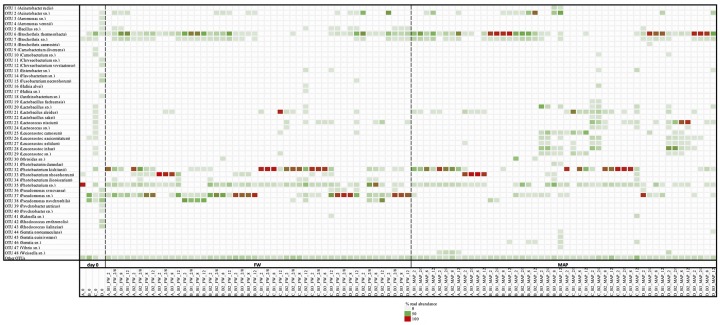
Heatmap of relative read abundance at species level for all samples (expressed in %) among the different storage conditions. Only the most abundant OTUs obtained in this study are specially indicated (>1%). Others OTUs are gathered in “Others OTUs.” Legend: food companies (A–D), with three batches each (B1, B2, B3), analyzed at the first (0) and the last day of storage, in food wrap (FW) and modified atmosphere (MAP) packaging. Temperature of storage: 2°C (2), 8°C (8), 12°C (12), and for a third of the shelf life at 2°C and for the rest of the shelf life at 8°C (2/8).

At this time, the major OTUs groups (Figure 5) are therefore different according to the food packaging: *Brochothrix thermosphacta*, *Lactobacillus algidus*, *Photobacterium kishitanii*, *Photobacterium phosphoreum*, *Pseudomonas psychrophila*, and *Pseudomonas* sp. are dominant in FW. While it concerns *Acinetobacter* sp., *Brochothrix thermosphacta*, *Lactobacillus algidus*, *Lactococcus piscium*, *Leuconostoc inhae*, *Leuconostoc gelidum*, *Leuconostoc* sp., *Photobacterium kishitanii*, *Photobacterium phosphoreum*, and *Pseudomonas* sp. in MAP.

### Variability of the Minced Pork Meat Ecosystem Between Samples

**FIGURE 6:**
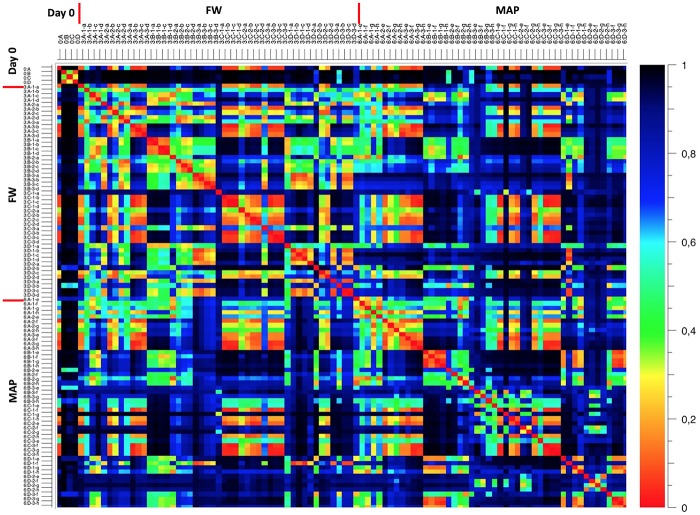
Global microbial dissimilarity obtained by metagenetics between samples for different conditions of storage. The heatmap shows the Bray-Curtis dissimilarity measure based on relative abundance of OTUS (genus scale). Values are given in dissimilarity counts (1 = 100% dissimilar, 0 = 0% dissimilar). Legend: analysis at day 0 (0), at day 3 (3) and at day 6 (6); food companies (A–D); three batches each (1, 2, 3); in food wrap (FW) and in modified atmosphere (MAP) packaging. Temperature of storage: 2°C in FW (A), for a third of the shelf life at 2°C and for the rest of the shelf life at 8°C in FW (B), 8°C in FW (C), 12°C in FW (D), 2°C in MAP (E), for a third of the shelf life at 2°C and for the rest of the shelf life at 8°C in MAP (F), 8°C in MAP (G), 12°C in MAP (H).

Genus relative abundance shows a high Bray-Curtis dissimilarity during the storage, and between the food companies and batches (Figure 6).

At day 0, samples showed a high dissimilarity (>70%) with the metadata groupings at the end of the shelf life. At this time, the food company A seems not to shared OTUs in common with the three others food industries.

At the end of the shelf life, Bray-Curtis index seems indicating that a relative similarity exists for OTUs contained within food companies A and C, and within B and D. This index also indicates a relative similarity concerning the temperature of storage, except for the industry D.

A synthetic view about the Bray-Curtis index between samples according to the food origin and storage condition is summarized in Table 3.

**TABLE 3:** Dominant bacteria represented in minced pork meat samples according to storage conditions.

Food companies	First day of storage	Last day of storage		
		
		Batch	FW	MAP
A	*Photobacterium* sp., *Ph. phosphoreum*	1	*Brochothrix thermosphacta*, *Photobacterium kishitanii*, *Pseudomonas* sp.	*B. thermosphacta*, *Ph. kishitanii*
		2	*B. thermosphacta*, *Ph. kishitanii*, *Pseudomonas* sp.	*B. thermosphacta*, *Ph. kishitanii*, *Weissella* sp.
		3	*Ph. phosphoreum*, *Pseudomonas* sp.	*Ph. phosphoreum*
B	*Pseudomonas* sp., *Ps. psychrophila*	1	*B. thermosphacta*, *Ps. psychrophila*	*B. thermosphacta*, *Ps. psychrophila*
		2	*B. thermosphacta*, *Photobacterium* sp., *Pseudomonas* sp.	*Acinetobacter* sp., *B. thermosphacta*, *Photobacterium* sp.
		3	*Ph. kishitanii*, *Ph. phosphoreum*, *Pseudomonas* sp.	*Acinetobacter* sp., *Lactobacillus* sp., *Leuconostoc* sp., *Ln. gelidum*, *Photobacterium* sp., *Ph. kishitanii*
C	*Photobacterium* sp., *Ph. kishitanii*	1	*Lactobacillus algidus*, *Ph. kishitanii*	*Lb. algidus*, *Ln. carnosum, Ln. inhae*, *Ph. kishitanii*
		2	*Photobacterium* sp., *Ph. kishitanii*, *Pseudomonas* sp., *Ps. phychrophila*	*Lb. algidus*, *Lactococcus piscium*, *Ln. inhae*, *Ph. kishitanii*
		3	*Ph. kishitanii*, *Pseudomonas* sp.	*Ph. kishitanii*
D	*Pseudomonas* sp., *Ps. psychrophila*, *Ps. syncyanea*	1	*B. thermosphacta*, *Pseudomonas* sp.,	*B. thermosphacta*, *Photobacterium* sp., *Pseudomonas* sp.
		2	*Acinetobacter* sp., *B. thermosphacta*, *Photobacterium* sp., *Ps. psychrophila*	*B. thermosphacta*, *Lc. piscium*, *Ln. gelidum, Ln. inhae*
		3	*Acinetobacter* sp., *Brochothrix* sp., *B. thermosphacta*, *Pseudomonas* sp.,	*B. thermosphacta*, *Ph. kishitanii*

*At species level, the taxa representing <20% in relative abundance were not considered as dominant in this table. FW (food wrap packaging), MAP (modified atmosphere packaging).*

## Discussion

In this study, we investigated the microbial spoilage community and dynamics of minced pork meat samples, among different conditions of production and food storage, using both 16S rRNA gene sequencing and classical microbiology. Indeed, whereas the dynamics of the bacterial community of meat and meat products have been studied before, Stoops et al. (2015) reported that little is known about differences in microbial changes during storage, and among the variability of batches production. Meat and meat products are highly perishable, with colonization and development of a great variety of microorganisms (Nychas et al., 2008; Pennacchia et al., 2009; Chaillou et al., 2015; Stellato et al., 2016; Garnier et al., 2017). The product composition (low/high pH, low/high concentration of glucose, water activity, …) and the storage conditions (temperature of storage and packaging conditions for example) may favor growth of microorganisms, that are responsible for the formation of spoilage (Argyri et al., 2015; Reid et al., 2017). This can lead to visible growth (slime, colonies), as textural changes, off-odors or off-flavors (Casaburi et al., 2014; Chaillou et al., 2015; Stoops et al., 2015; Del Blanco et al., 2017). In this context, minced meat is a potentially hazardous food product, vulnerable to bacterial spoilage, with a very short shelf life (Geeraerts et al., 2017) due to abundant and diverse substrate for bacterial growth and favorable growth conditions (Benson et al., 2014). In our study, the minced pork meat samples present a high water activity and a near-neutral pH which are in accordance with previous studies on this food matrix (Blixt and Borch, 2002; Andritsos et al., 2012).

The initial contamination of products, and also the initial level of lactic acid bacteria, is also a key factor that can influence the spoilage dynamic during storage (De Filippis et al., 2013). In our results, the microbial counts of the four manufacturers were quite different and psychrotrophic counts were higher for two food industries (Tables 1, 2). High levels of initial contamination in minced pork meat samples were also observed by Peruzy et al. (2019). This difference of the initial bacterial contamination is not in relation with the size of the company. These results can be explained by the fact that multiple sources of contamination can contribute to the initial composition of the meat microbiota (De Filippis et al., 2013), such as at the farm (hygiene practices, the conditions of animal transport, etc.) and at the slaughterhouse (automatic level of the process, cleaning practices, etc.). Initial carcass contamination can be also environmental, with contamination by tools, machines, and surfaces of slaughter equipment (Mann et al., 2016; Moretro et al., 2016). In addition, subsequent handling of meat in the operations of slicing, sectioning, portioning, and transferring in packages can determine further contamination in the handling points (Del Blanco et al., 2017).

The bacterial count at the end of the shelf life was over 7.0 log CFU/g, indicating that meat had probably begun to be deteriorated and would not be suitable for human consumption (Zhao et al., 2015). Indeed, it is generally recognized that microbial spoilage of meat occurs when counts reach arbitrary level between 7.0 log CFU/g (Nychas et al., 2008; Pothakos et al., 2014; Stoops et al., 2015; Reid et al., 2017; Spanu et al., 2018) and 8.00 log CFU/g (Nychas et al., 2008; Fall et al., 2012; Pothakos et al., 2014; Chaillou et al., 2015; Reid et al., 2017). However, these values are only indicative and refer here to the total viable count. Food spoilage needs to be assessed to the genus-species level, because potentially protective bacteria can also occur in food products.

As discussed by Del Blanco et al. (2017), common approaches for delaying meat spoilage and improving meat shelf life are available, including good hygienic practices and all the storage conditions. Among these, low storage temperatures and adequate packaging are considered as the most important factors (Koutsoumanis et al., 2006; Andritsos et al., 2012; Kaur et al., 2017). During the storage at 2°C, the arbitrary level of 7.0 log CFU/g was sometimes not reached. In addition, it can be observed that the microbial kinetics from 2 to 8°C were quite similar to those at 8°C, as described by Cauchie et al. (2017).

In relation with the food packaging, the most common used in meat and meat products are vacuum packaging and modified atmosphere packaging (MAP) (Caryé et al., 2005; Koutsoumanis et al., 2008; Dalcanton et al., 2013; Chaix et al., 2015; Silbande et al., 2016). In this study, a food wrap (FW) and a MAP (30% CO_2_ – 70% O_2_) packaging are used. The composition of modified atmosphere systems can be an effective way to reduce the growth rate of spoilage aerobic organisms and modify the microbial ecology of the product. But their effectiveness strongly depends on the initial microbial contamination of raw materials, storage temperature, film permeability and the carbon dioxide concentration used (20–40% is commonly used to suppress microbial growth) (Simpson and Carevic, 2004; Rotabakk et al., 2006; Stoops et al., 2015; Guillard et al., 2016; Saraiva et al., 2016; Couvert et al., 2017). The carbon dioxide concentration was here theoretically sufficient to limit the microbial growth. However, the higher percentage of oxygen can also enhance the growth of aerobic microbial communities in our samples. Moreover, some bacteria are able to grow in variable food packaging, as *Photobacterium* which is CO_2_-tolerant (Dalgaard, 1995; Fuertez-Perez et al., 2019). Also, in accordance with Stoops et al. (2015), it can be observed a significant production of carbon dioxide. This production may be the reflect of the development of bacterial groups belonging to lactic acid bacteria, *Brochothrix* or *Enterobacteriaceae* (Caryé et al., 2005). As environment of slaughtering and processing steps (Stellato et al., 2016), packaging materials can also be a source of contamination because they are not sterile in study. Further studies based on microbial contamination of food trays would also be interesting.

According to this, and based on the study by Stoops et al. (2015), viable counts are not suitable to characterize the microbial diversity of food products and to investigate thoroughly shifts in the bacterial communities during storage. Indeed, culture-dependent techniques largely underestimated the species richness and abundance. For a more detailed characterization of microbial communities in samples, originating from different ecological niches, a sequence-based approach was used, allowing identification of OTUs at various taxonomic levels (species, genus or family levels) (Stoops et al., 2015). However, without extensive studies involving a large number of samples under different storage conditions it will not be possible to determine exactly the bacterial ecosystem and the role of individual spoilage species (Pennacchia et al., 2011; Rouger et al., 2018). According to this, we analyzed minced meat samples from four different food companies, with three different batches per industries. In addition to previous studies based on the microbial description of minced meat samples (Stoops et al., 2015; Peruzy et al., 2019), our study aims to understand and monitor microbial dynamics and variability between food companies and food batches, according to different storage conditions.

In our results, the observed microbial diversity was relatively high, and the most abundant bacteria differ among samples. As observed by Stoops et al. (2015) in minced meat samples, an increase of microbial counts is coinciding with a decrease in bacterial diversity during storage. At the end of the storage period, the major genus taxa are represented by *Pseudomonas* in FW and *Brochothrix* in MAP. But it can also be observed a high diversity between food companies and batches (Table 3). Our results are in accordance with Peruzy et al. (2019), which also observed a dominance of the genus *Pseudomonas*, *Brochothrix*, and *Carnobacterium* in minced pork meat samples. Moreover, these results are not surprising because the microbial populations of refrigerated meat and pork-meat products are mainly composed by *Pseudomonas* spp., cold tolerant *Enterobaceriaceae*, lactic acid bacteria (such as *Lactobacillus* spp., *Lactococcus* spp., *Leuconostoc* spp., *Carnobacterium* spp., etc.), *Brochothrix thermosphacta*, *Clostridium* spp. (Koort et al., 2005; Liu et al., 2006; Nychas et al., 2008; Pennacchia et al., 2009, 2011; Casaburi et al., 2014; Stellato et al., 2016; Del Blanco et al., 2017; Geeraerts et al., 2017) and *Weissella* spp. (Pothakos et al., 2014; Stellato et al., 2016). Other genera isolated frequently from fresh pork meats are *Acinetobacter* spp., *Aeromonas* spp., *Enterococcus* spp., and *Moraxella* spp. (Zhao et al., 2015; Mann et al., 2016). However, these results are not completely in accordance with Stoops et al. (2015) because this study mentioned that *Lactobacillus algidus* and *Leuconostoc* sp. became the dominant bacteria in minced meat samples stored at 5°C under modified atmosphere (66% O2, 25% CO2, and 9% N2). These differences can be explained by different meat compositions (beef in the study by Stoops et al. (2015) and pork in our study), the initial contamination of samples, and the gas mixture used.

The results also showed the interest of using culture-independent method to better understand the changes of food microbiota over time, and in each food companies, according to the storage conditions. Indeed, metagenetics approach produce a large amount of data in a very short time (Cocolin et al., 2018; Den Besten et al., 2018), allowing to interpret and use these data to help agri-food companies in their decisions regarding food safety and quality decisions. Moreover, all the OTUs-species described as potentially spoilers in our study are well described in the literature (Table 4), and in minced pork meat samples (Stoops et al., 2015; Peruzy et al., 2019). The bacterial species present in our samples are also able to grow in meat matrices, and they are potentially responsible of spoilage effects, which can affect color, flavor, visual aspect, etc. (Pothakos et al., 2015). Sensory analyses would be interesting in this context, but were not performed in this study. Moreover, the enzymatic decarboxylation of amino acids, or the transamination of aldehydes and ketones, by bacteria results in the formation and accumulation of biogenic amines (BAs) (Jastrzębska et al., 2016). Biogenic amines (e.g., b-phenylethylamine, cadaverine, histamine, putrescrine, spermidine, spermine, tyramine and tryptamine) are reported in various foods including meat, fish, cheese, and wine (Papageorgiou et al., 2018). They can have health implications, such as allergic reactions, but also contribute to spoilage due to their putrid aroma (Stanborough et al., 2017). Therefore, as proposed by Cheng et al. (2016), the sum of BAs could be used as an indicator of pork meat quality and freshness during storage. Li et al. (2014) also showed that some BAs could be used as spoilage indicators of chilled pork.

**TABLE 4:** Examples of some microbial species occurring during chilled storage of meat and their potential spoilage effects.

Bacteria	Growth conditions	Spoilage effects	References
*Actinetobacter* spp.	Especially present in dairy and seafood products.	Low spoilage potential but can enhanced the growth of other spoilage bacteria by means of quorum sensing.	Pinu, 2016; Ghasemi-Varnamkhasti et al., 2018; Odeyemi et al., 2018; Hahne et al., 2019
*Brochothrix* spp.	In different gas composition, such as under air, modified atmosphere and vacuum-packaging. More tolerant in oxygen-depleted and CO2-enriched environments.	Sour, acid and cheesy odor.	Koutsoumanis et al., 2008; Nychas et al., 2008; Ercolini et al., 2011; Doulgeraki et al., 2012; Zhao et al., 2015; Mann et al., 2016; Del Blanco et al., 2017; Reid et al., 2017; Mansur et al., 2019
*Carnobacterium* spp.	In all types of packaging conditions. Predominance in low O_2_ packaging.	Spoilage effect can vary, producing volatile molecules with low sensory impacts (fruity or fermented odors, …)	Casaburi et al., 2011; Doulgeraki et al., 2012; Pothakos et al., 2015
*Lactobacillus* spp. (*Lb.* sakei, *Lb. fuchuensis*, *Lb. plantarum*, *Lb. curvatus*, *Lb. algidus*, *Lb. oligofermentans*, …)	In all types of packaging conditions. Predominance with high concentration of CO_2_.	Severe acidification, emission of off-odor compounds and ropy slime. However, lactic acid bacteria may produce lactic acid, which inhibits the growth of other families of bacteria. And some species can produce bacteriocins.	Kato et al., 2000; Fadda et al., 2010; Doulgeraki et al., 2012; Dalcanton et al., 2013; Nieminen et al., 2015; Pothakos et al., 2015; Zhao et al., 2015; Alvarez-Sieiro et al., 2016; Mann et al., 2016; Woraprayote et al., 2016; Stefanovic et al., 2017; Mansur et al., 2019
*Lactococcus* spp.	In various types of packaging.	Traditionally they have not been considered as spoilage microorganisms, but the spoilage potential of these bacteria is still scarcely known.	Kato et al., 2000; Doulgeraki et al., 2012; Rahkila et al., 2012; Dalcanton et al., 2013; Pothakos et al., 2014; Zhao et al., 2015; Mann et al., 2016; Mansur et al., 2019
*Leuconostoc* spp. (*Ln. gelidum*, *Ln. carnosum*, *Ln. mesenteroides*, …)	Under aerobic, vacuum and modified atmosphere packaging. Predominance with high concentration of O_2_.	Buttery aroma, formation of slime, blowing of packages, green discoloration.	Kato et al., 2000; Doulgeraki et al., 2012; Dalcanton et al., 2013; Nieminen et al., 2015; Pothakos et al., 2015; Zhao et al., 2015; Mann et al., 2016; Mansur et al., 2019
*Photobacterium* spp.	Under air, vacuum and modified atmosphere packaging. More frequently present in seafood products.	Typically not associated with spoilage of meat. Responsible for reducing TMAO to TMA, off-odor (produce volatile organic compounds) and biogenic amine formation. The mechanism underlying spoilage has not been clarified.	Nieminen et al., 2016; Li et al., 2019
*Pseudomonas* spp.	In different gas composition, such as under air, modified atmosphere and vacuum-packaging. Predominance under aerobic low temperature. Limitation in the bacterial flora by the presence of CO_2_ and/or the limitation of O_2_ in MAP packaging.	Slime, discoloration, off-odor producing.	Koutsoumanis et al., 2008; Nychas et al., 2008; Ercolini et al., 2011; Andritsos et al., 2012; Doulgeraki et al., 2012; Zhao et al., 2015; Mann et al., 2016; Del Blanco et al., 2017; Reid et al., 2017; Liu et al., 2018; Spanu et al., 2018; Mansur et al., 2019
*Weissella* spp.	Some can be found in salted and fermented foods. Present in vacuum packaging.	Greenish appearance. Can plays an important role in the fermentation process. Some species can produce bacteriocins.	Pothakos et al., 2015; Martins et al., 2016; Kim et al., 2017; Kariyawasam et al., 2019

However, it is important to add that some bacteria can be considered as protective, such as some lactic acid bacteria. As mentioned by Singh (2018), the presence of high LAB communities does not necessarily result in quality defect, and their intra-species variation to cause spoilage has already been recognized (Pothakos et al., 2015).

In the present study, we designed a method to collect MPM samples in order to explore the bacterial communities and diversity among different food origin and storage conditions. Indeed, the modification of the composition of the spoilage flora during storage is an important factor in assessing food quality (Holm et al., 2013). Although the bacteria consistently dominated the microbiota of MPM samples are known, results indicated that bacterial diversity needs to be addressed on the level of food companies and batches variations. As discussed by Rouger et al. (2017), it is important to overcome variability to better understand the factors underlying the diversity of spoilage bacterial communities, by (i) defining reproducible and reliable experimental conditions to lead to biological interpretation, or (ii) to multiplying sampling or experiments to obtain statistical significance of the results (Chaillou et al., 2015; Rouger et al., 2017). A seasonal effect on the microbial quality of minced meat has also been reported by Andritsos et al. (2012). In this paper, no conclusions about bacterial ecosystems for others food companies, or for different times of the year, should be dawn. Further data are so needed to determine diversity of spoilage microbiota in minced pork meat samples, according to others food industries, sampling periods and storage conditions. Also, a comparative evaluation of spoilage-related bacterial species and metabolic profiles, with growth parameters of these potentially spoilage bacteria in samples, will be studied in another study.

In conclusion, the combination of both culture-dependent and culture-independent analyses enabled us to explore the microbial communities of minced pork meat samples under different food origin and storage conditions, as previously described by Stoops et al. (2015). In our study, microbial changes during storage were monitored, according to a sampling in four food companies and for several batches. In accordance with previous studies we found that *Pseudomonas* and *Brochothrix* dominate the community at the end of the shelf life in FW and MAP, respectively, together with *Photobacterium*. The major OTUs groups are also often associated with pork meat spoilage in the scientific literature. And these results are also in accordance with studies conducted on the microbiota of minced meat by Stoops et al. (2015) and Peruzy et al. (2019). Psychrophilic spoilers dominated the microbiota of our samples, but each sample harbored a unique pork meat microbiota, depending on the manufacturing batch and the packaging used. The gas mixture and the temperature condition used in this study are probably the most important factors implied to the dynamics of the bacterial community. Further researches on the main contamination during slaughter production process, such as importance of processing environment, procedures and storage conditions, are desirable to provide a complete assessment of the microbiome of minced meat and to limit incidents of unexpected spoilage.

## Data Availability Statement

All biosample raw reads were deposited at the National Center for Biotechnology Information (NCBI) and are available under the BioProject ID PRJNA551357.

## Author Contributions

EC did the experiments, interpreted the results and wrote the manuscript. LD performed the experiments, supervised analyses and revised the manuscript. BT, PF, FF, GB, and GD were involved in the design of the study and provided help for interpretation of the results. AT and SB participated to the experiments. NK participated to the design of the study, interpretation of the results and writing of the manuscript. All authors read and approved the final manuscript.

## Conflict of Interest

PF and SB (Quality Partner sa, Liège, Belgium) were employed by the Department of Food Sciences (Faculty of Veterinary Medicine, University of Liège, Liège, Belgium) to perform 16S rRNA gene amplicon sequencing. The remaining authors declare that the research was conducted in the absence of any commercial or financial relationships that could be construed as a potential conflict of interest.

## Footnotes

1 http://microbiomeutil.sourceforge.net/#A_CS

2 http://www.arb-silva.de/

3 http://www.mothur.org

4 http://www.explicet.org

5 https://www.mybiosoftware.com/stamp-2-0-0-analyze-metagenomic-profiles.html
