# Supplementary material for: Assessment of Spoilage Bacterial Communities in Food Wrap and Modified Atmospheres-Packed Minced Pork Meat Samples by 16S rDNA Metagenetic Analysis
Source: Front Microbiol. 2020 Jan 21;10:3074. doi: 10.3389/fmicb.2019.03074 (PMC6985204; doi:10.3389/fmicb.2019.03074)
Supplement: Supplementary file 1 [file Data_Sheet_1.docx]

Supplementary Material

## Supplementary Figures

**Supplementary Figure 1.** Schematic representation of the methods used. Legend: FW (food wrap packaging) and MAP (modified atmosphere packaging).

**
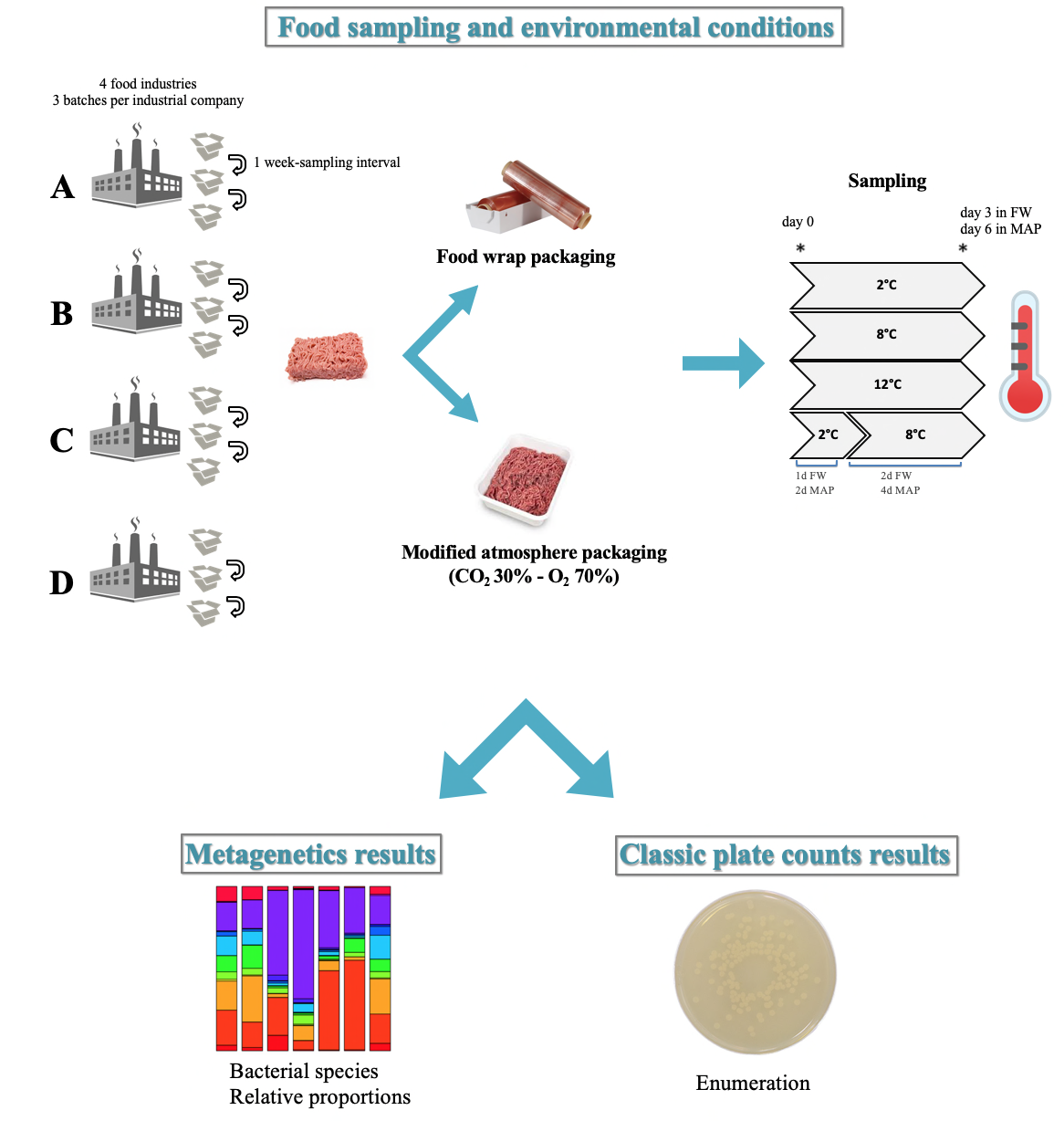
**

**Supplementary Figure 2.** Rarefaction curves for all samples (food companies A, B, C and D), based on an operational taxonomic unit (OTU) definition of 97% similarity (0.03 16S rRNA distance).

**
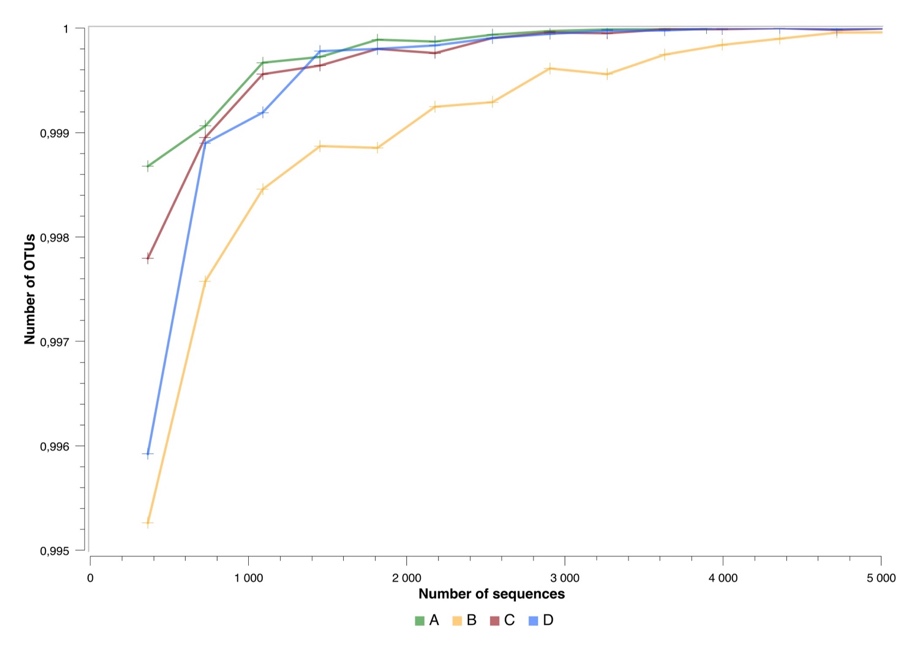
**

**Supplementary Figure 3.** Principal component analysis for 16S rRNA gene sequence data in food wrap (FW) and modified atmosphere (MAP) packaging, among different origin (food companies and batches) and storage temperature.

**
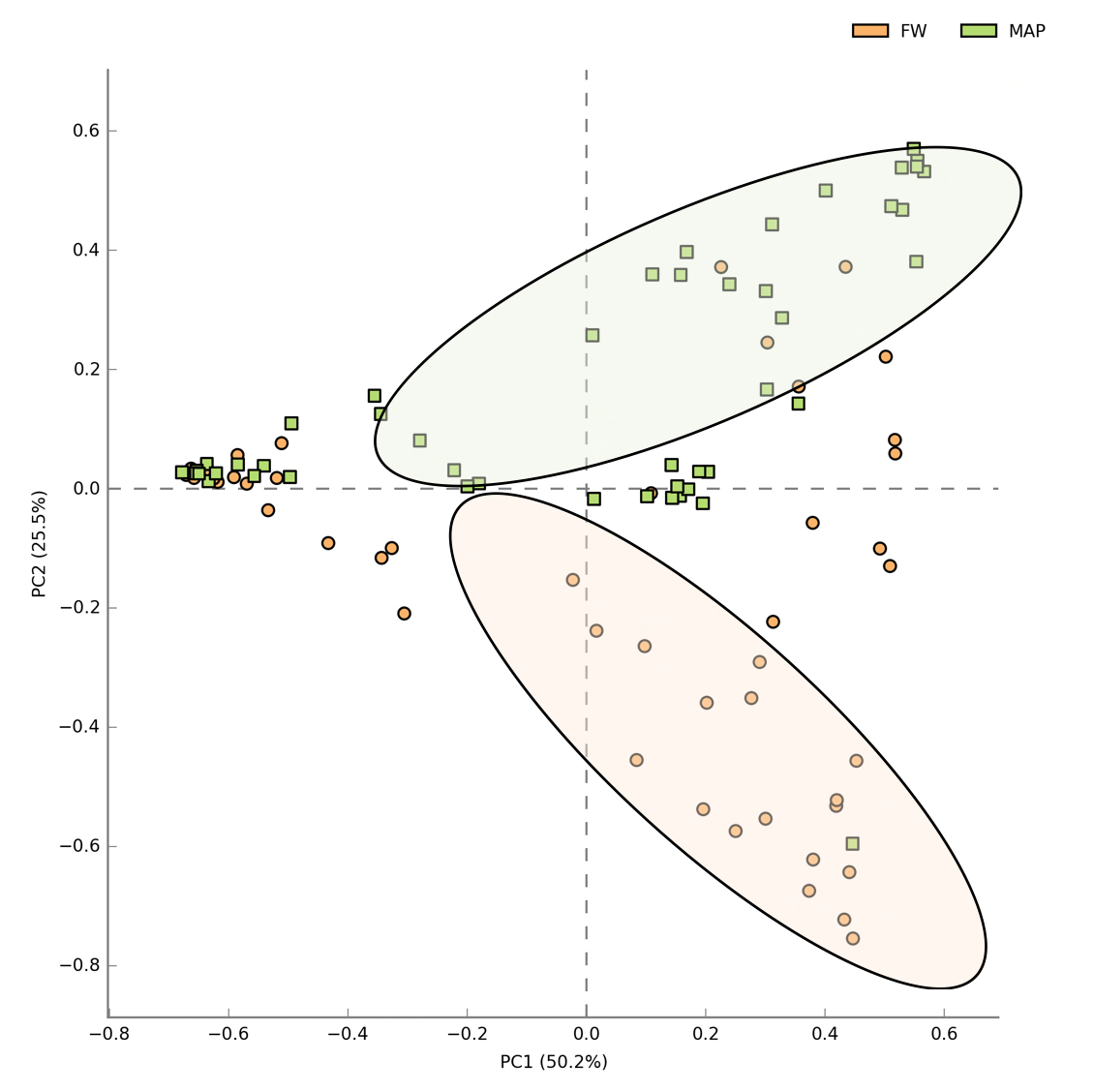
**

1. **Supplementary Tables**

**Supplementary Table 1.** Alpha diversity from metagenetics analysis at day 0.

| **Samples** | | **No. of OTUs** | **Coverage (%)** | **Inv. Simpson** | **Chao richness** | **Simpson evenness** |
| --- | --- | --- | --- | --- | --- | --- |
|  | A1 | 8 | 99.98 | 1.33 | 8.00 | 0.17 |
|  | A2 | 7 | 99.98 | 1.12 | 7.00 | 0.16 |
|  | A3 | 6 | 99.98 | 1.25 | 6.00 | 0.21 |
|  | B1 | 3 | 98.96 | 1.84 | 3.00 | 0.61 |
|  | B2 | 5 | 98.86 | 1.30 | 5.00 | 0.26 |
|  | B3 | 5 | 98.95 | 2.32 | 5.00 | 0.46 |
|  | C1 | 8 | 97.80 | 2.18 | 8.50 | 0.27 |
|  | C2 | 9 | 97.62 | 5.86 | 9.50 | 0.65 |
|  | C3 | 4 | 98.98 | 1.29 | 4.00 | 0.32 |
|  | D1 | 5 | 100.00 | 1.45 | 5.00 | 0.29 |
|  | D2 | 11 | 94.06 | 4.80 | 26.00 | 0.44 |
|  | D3 | 15 | 93.00 | 4.90 | 36.00 | 0.33 |

Legend: food companies (A, B, C, D), with three batches each (1, 2, 3).

**Supplementary Table 2.** Alpha diversity from metagenetics analysis at the end of the shelf life in food wrap packaging.

| **Samples** | | **No. of OTUs** | **Coverage (%)** | **Inv. Simpson** | **Chao richness** | **Simpson evenness** |
| --- | --- | --- | --- | --- | --- | --- |
|  | A1 a | 8 | 100.00 | 3.18 | 8.00 | 0.40 |
|  | A1 b | 7 | 100.00 | 2.35 | 7.00 | 0.34 |
|  | A1 c | 8 | 100.00 | 1.98 | 8.00 | 0.25 |
|  | A1 d | 8 | 100.00 | 1.13 | 7.00 | 0.24 |
|  | A2 a | 7 | 99.95 | 1.23 | 7.50 | 0.18 |
|  | A2 b | 8 | 99.98 | 1.88 | 8.00 | 0.23 |
|  | A2 c | 7 | 100.00 | 2.33 | 7.00 | 0.33 |
|  | A2 d | 9 | 100.00 | 2.25 | 9.00 | 0.25 |
|  | A3 a | 5 | 99.98 | 1.05 | 5.00 | 0.21 |
|  | A3 b | 7 | 99.98 | 1.21 | 7.00 | 0.17 |
|  | A3 c | 6 | 100.00 | 1.36 | 6.00 | 0.23 |
|  | A3 d | 7 | 99.95 | 2.67 | 8.00 | 0.38 |
|  | B1 a | 3 | 98.95 | 2.06 | 3.00 | 0.69 |
|  | B1 b | 3 | 98.95 | 1.94 | 3.00 | 0.65 |
|  | B1 c | 4 | 97.98 | 2.01 | 5.00 | 0.50 |
|  | B1 d | 4 | 98.96 | 2.15 | 4.00 | 0.54 |
|  | B2 a | 3 | 100.00 | 2.68 | 3.00 | 0.89 |
|  | B2 b | 4 | 100.00 | 2.56 | 4.00 | 0.64 |
|  | B2 c | 7 | 97.78 | 3.00 | 8.00 | 0.43 |
|  | B2 d | 5 | 100.00 | 2.02 | 5.00 | 0.40 |
|  | B3 a | 3 | 100.00 | 1.26 | 3.00 | 0.42 |
|  | B3 b | 4 | 98.81 | 1.59 | 4.00 | 0.40 |
|  | B3 c | 6 | 97.80 | 1.85 | 6.50 | 0.31 |
|  | B3 d | 4 | 98.85 | 1.13 | 4.00 | 0.28 |
|  | C1 a | 3 | 98.96 | 1.14 | 3.00 | 0.38 |
|  | C1 b | 3 | 97.89 | 1.04 | 4.00 | 0.35 |
|  | C1 c | 3 | 97.94 | 1.04 | 4.00 | 0.35 |
|  | C1 d | 4 | 98.96 | 1.36 | 4.00 | 0.34 |
|  | C2 a | 7 | 95.60 | 1.50 | 13.00 | 0.21 |
|  | C2 b | 6 | 97.62 | 1.22 | 6.25 | 0.20 |
|  | C2 c | 5 | 96.47 | 1.13 | 6.50 | 0.23 |
|  | C2 d | 8 | 97.96 | 2.23 | 9.00 | 0.28 |
|  | C3 a | 3 | 97.85 | 1.04 | 4.00 | 0.35 |
|  | C3 b | 4 | 98.94 | 1.17 | 4.00 | 0.29 |
|  | C3 c | 4 | 97.87 | 1.14 | 5.00 | 0.29 |
|  | C3 d | 9 | 95.70 | 2.55 | 11.00 | 0.28 |
|  | D1 a | 5 | 97.85 | 1.38 | 6.00 | 0.28 |
|  | D1 b | 4 | 96.59 | 1.07 | 7.00 | 0.27 |
|  | D1 c | 5 | 97.80 | 1.29 | 5.50 | 0.26 |
|  | D1 d | 11 | 95.74 | 3.30 | 13.00 | 0.30 |
|  | D2 a | 4 | 97.85 | 2.00 | 5.00 | 0.50 |
|  | D2 b | 3 | 100.00 | 1.85 | 3.00 | 0.62 |
|  | D2 c | 4 | 100.00 | 1.59 | 4.00 | 0.40 |
|  | D2 d | 5 | 98.96 | 1.87 | 5.00 | 0.37 |
|  | D3 a | 7 | 95.79 | 2.59 | 13.00 | 0.37 |
|  | D3 b | 4 | 98.98 | 1.59 | 4.00 | 0.40 |
|  | D3 c | 4 | 100.00 | 1.59 | 4.00 | 0.40 |
|  | D3 d | 5 | 98.90 | 1.72 | 5.00 | 0.34 |

Legend: food companies (A, B, C, D), three batches each (1, 2, 3). At different temperature of storage: 2°C (a), for a third of the shelf life at 2°C and for the rest of the shelf life at 8°C (b), 8°C (c), and 12°C (d).

**Supplementary Table 3.** Alpha diversity from metagenetics analysis at the end of the shelf life in modified atmosphere packaging.

| **Samples** | | **No. of OTUs** | **Coverage (%)** | **Inv. Simpson** | **Chao richness** | **Simpson evenness** |
| --- | --- | --- | --- | --- | --- | --- |
|  | A1 a | 8 | 100.00 | 2.52 | 8.00 | 0.32 |
|  | A1 b | 6 | 100.00 | 1.92 | 6.00 | 0.32 |
|  | A1 c | 9 | 100.00 | 1.74 | 9.00 | 0.19 |
|  | A1 d | 8 | 99.98 | 2.61 | 8.00 | 0.33 |
|  | A2 a | 8 | 100.00 | 1.25 | 8.00 | 0.16 |
|  | A2 b | 9 | 99.95 | 1.94 | 10.00 | 0.22 |
|  | A2 c | 8 | 100.00 | 2.55 | 8.00 | 0.32 |
|  | A2 d | 9 | 99.98 | 2.17 | 9.00 | 0.24 |
|  | A3 a | 7 | 100.00 | 1.40 | 7.00 | 0.20 |
|  | A3 b | 7 | 99.95 | 1.11 | 8.00 | 0.16 |
|  | A3 c | 7 | 100.00 | 1.09 | 7.00 | 0.16 |
|  | A3 d | 6 | 99.98 | 1.37 | 6.00 | 0.23 |
|  | B1 a | 4 | 97.85 | 1.42 | 5.00 | 0.35 |
|  | B1 b | 4 | 97.87 | 1.14 | 5.00 | 0.29 |
|  | B1 c | 7 | 97.98 | 1.27 | 7.25 | 0.18 |
|  | B1 d | 5 | 97.92 | 1.14 | 5.33 | 0.23 |
|  | B2 a | 6 | 98.91 | 2.89 | 6.00 | 0.48 |
|  | B2 b | 5 | 98.90 | 2.10 | 5.00 | 0.42 |
|  | B2 c | 5 | 100.00 | 2.56 | 5.00 | 0.51 |
|  | B2 d | 7 | 97.96 | 2.08 | 7.33 | 0.30 |
|  | B3 a | 5 | 98.98 | 2.53 | 5.00 | 0.51 |
|  | B3 b | 5 | 98.97 | 2.18 | 5.00 | 0.44 |
|  | B3 c | 6 | 96.74 | 2.70 | 9.00 | 0.45 |
|  | B3 d | 9 | 97.96 | 3.66 | 9.50 | 0.41 |
|  | C1 a | 4 | 98.95 | 1.11 | 4.00 | 0.28 |
|  | C1 b | 4 | 97.92 | 2.03 | 5.00 | 0.51 |
|  | C1 c | 4 | 98.96 | 1.59 | 4.00 | 0.40 |
|  | C1 d | 5 | 97.96 | 2.56 | 6.00 | 0.51 |
|  | C2 a | 5 | 98.96 | 3.22 | 5.00 | 0.64 |
|  | C2 b | 8 | 97.78 | 4.06 | 8.33 | 0.51 |
|  | C2 c | 5 | 98.95 | 1.36 | 5.00 | 0.27 |
|  | C2 d | 3 | 98.77 | 1.22 | 3.00 | 0.41 |
|  | C3 a | 2 | 98.96 | 1.02 | 2.00 | 0.51 |
|  | C3 b | 3 | 98.98 | 1.09 | 3.00 | 0.36 |
|  | C3 c | 3 | 98.97 | 1.11 | 3.00 | 0.37 |
|  | C3 d | 10 | 94.81 | 4.95 | 16.00 | 0.50 |
|  | D1 a | 4 | 100.00 | 1.41 | 4.00 | 0.35 |
|  | D1 b | 5 | 97.87 | 1.37 | 6.00 | 0.27 |
|  | D1 c | 6 | 96.91 | 1.71 | 9.00 | 0.28 |
|  | D1 d | 4 | 98.95 | 1.88 | 4.00 | 0.47 |
|  | D2 a | 7 | 97.94 | 2.44 | 8.00 | 0.35 |
|  | D2 b | 5 | 100.00 | 1.99 | 5.00 | 0.40 |
|  | D2 c | 5 | 98.96 | 1.83 | 5.00 | 0.37 |
|  | D2 d | 5 | 100.00 | 1.49 | 5.00 | 0.30 |
|  | D3 a | 4 | 98.90 | 1.17 | 4.00 | 0.29 |
|  | D3 b | 4 | 98.91 | 1.12 | 4.00 | 0.28 |
|  | D3 c | 4 | 98.99 | 1.43 | 4.00 | 0.36 |
|  | D3 d | 8 | 98.90 | 2.84 | 8.00 | 0.35 |

Legend: food companies (A, B, C, D), three batches each (1, 2, 3). At different temperature of storage: 2°C (a), for a third of the shelf life at 2°C and for the rest of the shelf life at 8°C (b), 8°C (c), and 12°C (d).

**Supplementary Table 4.** Distribution of metagenetic reads percentages at Genus level for each food companies, at day 0.

| **Samples** | ***Aeromonas*** | ***Brochothrix*** | ***Carnobacterium*** | ***Chryseobacterium*** | ***Fusobacterium*** | ***Lactococcus*** | ***Leuconostoc*** | ***Photobacterium*** | ***Pseudomonas*** | ***Rhodococcus*** | **Others** |
| --- | --- | --- | --- | --- | --- | --- | --- | --- | --- | --- | --- |
| A | 0.00 | 1.35 | 0.00 | 0.00 | 0.00 | 0.00 | 0.00 | 86.77 | 0.00 | 0.00 | 11.88 |
| B | 0.00 | 5.20 | 0.00 | 0.00 | 0.00 | 0.00 | 0.00 | 0.00 | 38.71 | 0.00 | 56.09 |
| C | 0.00 | 1.76 | 10.08 | 0.00 | 0.00 | 9.50 | 6.85 | 19.98 | 0.00 | 0.00 | 51.84 |
| D | 6.45 | 6.03 | 0.00 | 8.25 | 13.47 | 0.00 | 0.00 | 0.00 | 25.67 | 11.27 | 28.86 |

At Genus levels, the taxa representing <5% in relative abundance were merged in the category of “Others”.

**Supplementary Table 5.** Distribution of metagenetic reads percentages at Genus level during cold storage of minced pork meat in a food wrap packaging.

| **Samples** | ***Acinetobacter*** | ***Brochothrix*** | ***Lactobacillus*** | ***Lactococcus*** | ***Leuconostoc*** | ***Photobacterium*** | ***Pseudomonas*** | **Others** |
| --- | --- | --- | --- | --- | --- | --- | --- | --- |
| A1 a | 0.77 | 2.32 | 0.21 | 0.00 | 0.02 | 78.54 | 8.63 | 9.51 |
| A1 b | 5.56 | 15.66 | 0.15 | 0.00 | 0.08 | 40.25 | 32.24 | 6.06 |
| A1 c | 2.81 | 58.26 | 0.27 | 0.00 | 0.10 | 18.56 | 15.43 | 4.57 |
| A1 d | 5.43 | 63.23 | 0.30 | 0.33 | 0.12 | 1.92 | 22.30 | 6.37 |
| A2 a | 0.02 | 4.24 | 0.04 | 0.11 | 0.00 | 84.76 | 4.24 | 6.59 |
| A2 b | 0.11 | 8.39 | 0.07 | 0.18 | 0.07 | 66.04 | 8.39 | 16.76 |
| A2 c | 0.00 | 15.81 | 0.41 | 0.11 | 0.07 | 22.91 | 15.81 | 44.88 |
| A2 d | 0.05 | 20.33 | 0.17 | 0.07 | 0.07 | 17.67 | 20.33 | 41.30 |
| A3 a | 0.00 | 1.52 | 0.56 | 0.00 | 0.02 | 94.94 | 0.29 | 2.66 |
| A3 b | 0.00 | 5.97 | 1.81 | 0.18 | 0.02 | 87.33 | 0.71 | 3.99 |
| A3 c | 0.00 | 10.55 | 2.12 | 0.09 | 0.00 | 81.40 | 1.61 | 4.23 |
| A3 d | 0.00 | 16.79 | 0.29 | 0.23 | 0.03 | 36.72 | 40.32 | 5.62 |
| B1 a | 0.34 | 44.47 | 0.09 | 0.04 | 0.04 | 0.69 | 49.57 | 4.76 |
| B1 b | 0.31 | 58.73 | 0.13 | 0.20 | 0.33 | 1.40 | 34.99 | 3.90 |
| B1 c | 0.54 | 59.15 | 0.07 | 0.07 | 0.02 | 1.32 | 38.22 | 0.60 |
| B1 d | 0.62 | 45.72 | 0.18 | 0.13 | 0.18 | 2.02 | 47.33 | 3.82 |
| B2 a | 0.36 | 15.70 | 0.10 | 0.10 | 0.36 | 29.40 | 42.62 | 11.37 |
| B2 b | 3.59 | 22.00 | 0.25 | 0.22 | 0.05 | 15.03 | 52.21 | 6.65 |
| B2 c | 4.49 | 24.16 | 0.61 | 0.08 | 0.15 | 11.47 | 45.33 | 13.71 |
| B2 d | 1.81 | 58.23 | 0.02 | 0.15 | 0.05 | 23.54 | 3.23 | 12.96 |
| B3 a | 0.19 | 2.84 | 0.11 | 0.06 | 0.38 | 6.15 | 72.03 | 18.25 |
| B3 b | 0.49 | 3.30 | 0.21 | 0.15 | 0.83 | 15.47 | 65.25 | 14.30 |
| B3 c | 1.93 | 2.56 | 0.53 | 0.10 | 0.99 | 20.02 | 63.83 | 10.03 |
| B3 d | 1.40 | 2.19 | 0.24 | 0.02 | 0.39 | 1.77 | 81.54 | 12.44 |
| C1 a | 0.06 | 0.32 | 4.60 | 0.06 | 0.02 | 90.25 | 0.58 | 4.11 |
| C1 b | 0.04 | 0.42 | 0.94 | 0.15 | 0.06 | 92.66 | 0.69 | 5.04 |
| C1 c | 0.15 | 0.24 | 0.76 | 0.02 | 0.02 | 94.96 | 0.65 | 3.19 |
| C1 d | 0.02 | 1.16 | 82.22 | 0.07 | 7.11 | 6.13 | 0.31 | 3.00 |
| C2 a | 0.02 | 1.05 | 6.29 | 6.97 | 0.33 | 73.80 | 0.84 | 10.71 |
| C2 b | 0.18 | 1.63 | 2.49 | 1.16 | 0.51 | 76.34 | 1.70 | 16.00 |
| C2 c | 0.02 | 0.95 | 0.91 | 0.81 | 0.08 | 80.35 | 2.04 | 14.84 |
| C2 d | 0.03 | 1.06 | 5.26 | 2.86 | 0.31 | 63.43 | 17.52 | 9.53 |
| C3 a | 0.00 | 0.33 | 0.06 | 1.00 | 0.00 | 90.86 | 1.21 | 6.43 |
| C3 b | 0.00 | 0.62 | 0.27 | 3.74 | 0.00 | 86.66 | 1.98 | 6.73 |
| C3 c | 0.06 | 0.22 | 0.45 | 4.20 | 0.13 | 87.88 | 0.97 | 6.08 |
| C3 d | 0.12 | 1.07 | 0.92 | 3.73 | 0.12 | 32.10 | 49.12 | 12.82 |
| D1 a | 1.46 | 5.96 | 0.06 | 0.13 | 0.04 | 6.28 | 79.14 | 6.91 |
| D1 b | 0.13 | 1.43 | 0.02 | 0.11 | 0.04 | 0.77 | 85.27 | 12.22 |
| D1 c | 1.00 | 7.36 | 0.04 | 0.10 | 0.02 | 1.58 | 79.72 | 10.18 |
| D1 d | 1.01 | 37.49 | 3.17 | 2.24 | 3.05 | 6.81 | 36.19 | 10.05 |
| D2 a | 34.72 | 56.00 | 0.11 | 0.11 | 0.13 | 0.53 | 1.40 | 6.99 |
| D2 b | 0.11 | 2.01 | 0.06 | 0.30 | 0.37 | 60.81 | 27.75 | 8.59 |
| D2 c | 0.02 | 3.31 | 0.09 | 1.52 | 0.32 | 72.71 | 15.76 | 6.27 |
| D2 d | 0.02 | 1.69 | 0.10 | 15.93 | 0.80 | 9.05 | 68.10 | 4.30 |
| D3 a | 0.00 | 0.33 | 0.06 | 1.00 | 0.00 | 90.96 | 1.21 | 6.43 |
| D3 b | 0.00 | 0.62 | 0.27 | 3.74 | 0.00 | 86.66 | 1.98 | 6.73 |
| D3 c | 0.06 | 0.22 | 0.45 | 4.20 | 0.13 | 87.88 | 0.97 | 6.08 |
| D3 d | 0.12 | 1.07 | 0.92 | 3.73 | 0.12 | 32.10 | 49.12 | 12.82 |

At Genus levels, the taxa representing <5% in relative abundance were merged in the category of “Others”. Legend: food companies (A, B, C, D), three batches each (1, 2, 3). At different storage temperature: 2°C (a), for a third of the shelf life at 2°C and for the rest of the shelf life at 8°C (b), 8°C (c), and 12°C (d).

**Supplementary Table 6.** Distribution of metagenetic reads percentages at Genus level during cold storage of minced pork meat in a modified atmosphere packaging.

| **Samples** | ***Acinetobacter*** | ***Brochothrix*** | ***Enterobacter*** | ***Lactobacillus*** | ***Lactococcus*** | ***Leuconostoc*** | ***Myroides*** | ***Photobacterium*** | ***Pseudomonas*** | ***Serratia*** | ***Weissella*** | **Others** |
| --- | --- | --- | --- | --- | --- | --- | --- | --- | --- | --- | --- | --- |
| A1 a | 0.21 | 43.75 | 0.00 | 4.93 | 0.05 | 1.44 | 0.00 | 40.09 | 3.95 | 0.00 | 0.00 | 5.25 |
| A1 b | 0.08 | 58.92 | 0.00 | 2.06 | 0.00 | 0.59 | 0.00 | 28.89 | 0.47 | 0.00 | 0.00 | 8.99 |
| A1 c | 2.30 | 21.05 | 0.00 | 0.97 | 0.13 | 0.56 | 0.00 | 66.71 | 0.35 | 0.00 | 0.00 | 7.74 |
| A1 d | 22.06 | 52.19 | 0.00 | 2.02 | 0.02 | 1.02 | 0.00 | 18.55 | 0.37 | 0.00 | 0.00 | 3.69 |
| A2 a | 0.00 | 3.23 | 0.00 | 1.74 | 0.31 | 0.38 | 0.00 | 85.12 | 0.33 | 0.00 | 4.16 | 4.64 |
| A2 b | 0.02 | 18.31 | 0.00 | 0.17 | 0.07 | 3.11 | 0.00 | 68.27 | 0.37 | 0.00 | 9.12 | 0.52 |
| A2 c | 0.00 | 12.38 | 0.00 | 0.43 | 0.06 | 12.95 | 0.00 | 55.43 | 0.23 | 0.00 | 12.78 | 5.53 |
| A2 d | 2.40 | 57.40 | 0.00 | 0.42 | 0.03 | 1.19 | 0.00 | 34.41 | 0.32 | 0.00 | 2.43 | 1.23 |
| A3 a | 0.00 | 3.23 | 0.00 | 11.52 | 0.27 | 1.63 | 0.00 | 77.79 | 0.16 | 0.00 | 0.00 | 5.23 |
| A3 b | 0.00 | 2.53 | 0.00 | 1.60 | 0.07 | 0.72 | 0.00 | 91.85 | 0.02 | 0.00 | 0.00 | 3.19 |
| A3 c | 0.00 | 0.87 | 0.00 | 2.79 | 0.09 | 0.26 | 0.00 | 93.48 | 0.07 | 0.00 | 0.00 | 2.37 |
| A3 d | 0.00 | 13.91 | 0.00 | 1.25 | 0.07 | 0.18 | 0.00 | 81.58 | 0.02 | 0.00 | 0.00 | 2.99 |
| B1 a | 0.04 | 77.10 | 0.00 | 0.00 | 0.10 | 0.08 | 0.00 | 0.72 | 14.18 | 0.02 | 0.00 | 6.77 |
| B1 b | 0.13 | 87.70 | 0.00 | 0.15 | 0.21 | 0.92 | 0.00 | 0.61 | 3.96 | 0.10 | 0.06 | 5.94 |
| B1 c | 1.56 | 87.60 | 0.00 | 0.65 | 0.26 | 1.62 | 0.00 | 3.03 | 1.64 | 0.73 | 0.00 | 2.83 |
| B1 d | 1.98 | 90.02 | 0.00 | 0.41 | 0.18 | 0.46 | 0.00 | 0.68 | 1.47 | 2.20 | 0.00 | 2.25 |
| B2 a | 2.63 | 44.51 | 0.00 | 0.60 | 0.37 | 0.35 | 28.26 | 1.96 | 13.29 | 0.22 | 0.00 | 7.01 |
| B2 b | 2.57 | 55.77 | 0.00 | 0.30 | 0.00 | 0.27 | 0.05 | 29.19 | 2.30 | 0.99 | 0.00 | 8.21 |
| B2 c | 26.03 | 52.63 | 0.00 | 0.48 | 0.03 | 0.08 | 0.14 | 12.51 | 1.89 | 1.80 | 0.00 | 3.31 |
| B2 d | 63.24 | 25.60 | 0.00 | 0.27 | 0.03 | 0.09 | 3.38 | 0.39 | 2.40 | 1.99 | 0.00 | 0.90 |
| B3 a | 0.03 | 4.67 | 0.00 | 39.74 | 0.00 | 46.66 | 0.00 | 0.60 | 5.21 | 0.00 | 0.00 | 2.52 |
| B3 b | 0.05 | 1.31 | 0.00 | 10.99 | 0.00 | 62.29 | 0.00 | 19.57 | 2.67 | 0.08 | 0.00 | 2.54 |
| B3 c | 32.16 | 1.45 | 0.00 | 1.18 | 0.05 | 11.56 | 0.00 | 44.57 | 0.93 | 0.35 | 0.00 | 7.38 |
| B3 d | 47.49 | 5.56 | 0.00 | 7.06 | 0.19 | 13.60 | 0.00 | 8.61 | 2.16 | 11.25 | 0.00 | 1.56 |
| C1 a | 0.04 | 77.10 | 0.00 | 0.00 | 0.10 | 0.08 | 0.00 | 0.72 | 14.18 | 0.02 | 0.00 | 6.77 |
| C1 b | 0.13 | 87.70 | 0.00 | 0.15 | 0.21 | 0.92 | 0.00 | 0.61 | 3.96 | 0.10 | 0.06 | 5.94 |
| C1 c | 1.56 | 87.60 | 0.00 | 0.65 | 0.26 | 1.62 | 0.00 | 3.03 | 1.64 | 0.73 | 0.00 | 2.83 |
| C1 d | 1.98 | 90.02 | 0.00 | 0.41 | 0.18 | 0.46 | 0.00 | 0.68 | 1.47 | 2.20 | 0.00 | 2.25 |
| C2 a | 0.06 | 0.21 | 0.37 | 15.56 | 29.44 | 42.07 | 0.00 | 8.25 | 0.00 | 0.03 | 0.00 | 2.59 |
| C2 b | 0.00 | 0.36 | 1.13 | 30.15 | 18.85 | 27.30 | 0.00 | 2.40 | 0.16 | 0.58 | 0.00 | 9.48 |
| C2 c | 0.03 | 0.15 | 0.55 | 8.96 | 2.32 | 2.11 | 0.00 | 80.62 | 0.03 | 0.10 | 0.00 | 4.35 |
| C2 d | 0.03 | 0.43 | 0.06 | 6.76 | 1.30 | 0.35 | 0.00 | 72.89 | 0.00 | 0.00 | 0.00 | 17.54 |
| C3 a | 0.00 | 0.09 | 0.00 | 0.42 | 0.94 | 0.02 | 0.00 | 95.03 | 0.00 | 0.00 | 0.00 | 3.50 |
| C3 b | 0.00 | 0.05 | 0.00 | 1.12 | 2.88 | 0.11 | 0.00 | 94.04 | 0.00 | 0.00 | 0.00 | 1.71 |
| C3 c | 0.00 | 0.15 | 0.13 | 0.69 | 3.58 | 0.08 | 0.00 | 91.61 | 0.05 | 0.00 | 0.00 | 3.41 |
| C3 d | 0.81 | 1.49 | 8.59 | 11.51 | 9.68 | 0.60 | 0.00 | 29.12 | 0.55 | 4.75 | 0.00 | 24.06 |
| D1 a | 0.40 | 11.96 | 0.00 | 0.15 | 0.30 | 0.02 | 0.00 | 1.66 | 80.04 | 0.30 | 0.00 | 3.16 |
| D1 b | 0.05 | 80.42 | 0.00 | 1.19 | 0.38 | 8.49 | 0.00 | 0.28 | 4.34 | 0.00 | 0.00 | 3.57 |
| D1 c | 0.05 | 71.83 | 0.00 | 0.19 | 2.99 | 0.99 | 0.00 | 19.27 | 1.11 | 0.00 | 0.00 | 2.87 |
| D1 d | 13.51 | 66.96 | 0.00 | 0.20 | 0.11 | 0.31 | 0.00 | 1.00 | 13.14 | 0.02 | 0.00 | 4.31 |
| D2 a | 0.20 | 7.77 | 0.00 | 8.68 | 8.14 | 60.50 | 0.00 | 0.25 | 0.84 | 0.00 | 9.77 | 2.08 |
| D2 b | 0.05 | 2.45 | 0.00 | 1.53 | 26.90 | 62.02 | 0.00 | 1.55 | 0.18 | 0.00 | 0.05 | 5.19 |
| D2 c | 0.00 | 8.29 | 0.00 | 0.26 | 69.19 | 16.21 | 0.00 | 1.96 | 0.14 | 0.00 | 0.00 | 3.33 |
| D2 d | 0.00 | 4.47 | 0.00 | 1.94 | 78.91 | 7.77 | 0.00 | 4.08 | 0.07 | 0.00 | 0.02 | 2.60 |
| D3 a | 0.13 | 84.17 | 0.00 | 3.04 | 0.09 | 2.65 | 0.00 | 0.24 | 1.43 | 0.02 | 0.00 | 7.85 |
| D3 b | 0.09 | 87.19 | 0.00 | 1.12 | 0.15 | 1.78 | 0.00 | 1.72 | 0.26 | 0.02 | 0.00 | 7.52 |
| D3 c | 0.15 | 81.60 | 0.13 | 0.40 | 2.82 | 1.06 | 0.00 | 12.88 | 0.08 | 0.03 | 0.00 | 0.54 |
| D3 d | 16.00 | 51.43 | 2.04 | 5.61 | 4.83 | 0.37 | 0.00 | 6.15 | 1.02 | 4.32 | 0.00 | 7.99 |

At Genus levels, the taxa representing <5% in relative abundance were merged in the category of “Others”. Legend: food companies (A, B, C, D), three batches each (1, 2, 3). At different storage temperature: 2°C (a), for a third of the shelf life at 2°C and for the rest of the shelf life at 8°C (b), 8°C (c), and 12°C (d).
